# Assessing large language models as assistive tools in selecting first trial lens parameters for orthokeratology

**DOI:** 10.3389/fmed.2026.1741987

**Published:** 2026-02-02

**Authors:** Yijin Han, Junhan Wei, Jiaqi Wang, Yi-Ming Guo, Shaoguo Li, Lu Ye

**Affiliations:** Shaanxi Eye Hospital, Xi’an People’s Hospital (Xi’an Fourth Hospital), Affiliated People’s Hospital of Northwest University, Xi’an, Shaanxi, China

**Keywords:** ChatGPT, large language models, myopia, orthokeratology, refractive error

## Abstract

**Purpose:**

Large language models (LLMs) have the potential to be powerful tools in optometry. Orthokeratology is widely used in clinical interventions for myopia control. This study aims to evaluate the performance of LLMs as assistive tools in the CRT-related orthokeratology fitting workflow.

**Methods:**

This retrospective analysis used four LLMs (GPT-4o, GPT-o3, GPT-4.1 and Claude 3.7 Sonnet) to analyze refractive error cases and get responses regarding the parameters of the first trial lens. Subjective evaluation includes the accuracy and overall quality of the answers provided, and objective evaluation focuses on differences in the parameters of the first trial lens.

**Results:**

GQS and accuracy differed across models [χ^2^(3) = 39.85, *p* < 0.001; Kendall’s W = 0.148]. GPT-o3 and GPT-4o showed the strongest overall performance on the complete response (GQS: 4.66 ± 0.48 vs. 4.47 ± 0.5, Good ratings: 83.3% vs. 76.7%), For first trial lens parameters, feasibility errors decreased across the two correction rounds, LLM outputs showed tendencies concentrated in key fitting parameters, particularly a smaller BC radius (mm) and a larger RZD, while Bland–Altman analyses indicated that most observations lay within the 95% limits of agreement.

**Conclusion:**

LLMs may support routine CRT-related decision support. However, first trial-lens parameter selection required feasibility constraints and clinician verification, with systematic parameter bias mainly involving BC and RZD.

## 1 Introduction

Myopia is a significant public health issue with increasing in its prevalence and severity around the world. Interests and efforts in treatments for controlling myopia have been intensified, leading to a number of effective strategies into preventing and slowing the myopia progression in children (1–3). Since receiving Food and Drug Administration (FDA)’s approval for correcting refractive error in 2002, orthokeratology has been widely used for myopia correction and control in children and adolescents (4–7). These lenses are designed with reverse geometry to purposely flatten the cornea’s shape by applying gentle pressure to its center during sleep (8, 9). Among various designs, corneal refractive therapy (CRT) lenses from Paragon consist of three basic parameters: the base curve (BC), the returning zone depth (RZD) and the landing zone angle (LZA) (10, 11). Currently, the first CRT trial lens is typically selected using manufacturer-provided tools such as sliding card or application (APP), which are fast, standardized (12). However, these tools are largely one-way numeric systems and provide limited interactive explanation of fitting logic. In clinical practice fitting, doctors often need to understand why a particular parameter set is recommended, how multiple ocular variables jointly influence selection, and how to plan stepwise adjustments after the initial trial based on the fitting pattern. As software and machine-learning–assisted fitting continues to expand, the need for an interface that supports interactive clarification and reasoning alongside constraint-based recommendations becomes increasingly relevant.

Large language model (LLM) is a type of AI model using deep neural networks to learn the relationships between words in natural language, using large datasets of text to train (13). In recent years, research related to large language models in the medicine and healthcare field has been emerging in an endless stream (14), mainly focusing on clinical examination case analysis (15, 16), diagnostic decision-making (17–19), and patient guidance (20), education (21, 22) and care (23, 24). It has been reported that LLMs exhibited promising performance in providing disease diagnostic suggestions and patient care information across various ophthalmic domains, such as myopia, glaucoma, cataract, and retinal diseases, exhibiting high accuracy (25–31). However, despite the many advantages of LLMs, this does not mean that they can replace doctors. Instead, they can serve as valuable tools for ophthalmic medical professionals (32). Unlike traditional tools such as sliding card or APP, which rely on data or rigid instructions, LLMs could facilitate dynamic, conversational learning experiences and human-like cognitive abilities. This two-way communication provides practitioners with simulated training to help improve their initial decision-making abilities and patient interaction skills.

Currently, in the optometry research field, the combination of LLMs and myopia diagnosis and patient education is popular (27, 30, 33, 34). For example, ChatGPT-4o demonstrated 90% accuracy in guiding treatment decisions for pediatric refractive error, even when dealing with incomplete or abnormal data (26). However, with regard to the diagnosis of choosing orthokeratology interventions and selection of the first trial lens parameter programs, LLMs as a potential complementary tool to provide strategies have not yet been evaluated. Therefore, we evaluated four LLMs (ChatGPT-4.1, ChatGPT-4o, ChatGPT-o3, and Claude 3.7 Sonnet) using a consistent CRT-related query workflow. Performance was assessed using both subjective and objective measures to characterize their potential role and limitations under manufacturer constraints, with clinician verification remaining essential—particularly for first trial-lens parameter generation.

## 2 Materials and methods

### 2.1 Study design

Although the current literature reports the increasing use of Large Language Models (LLMs) in optometry, few studies have examined the quality of selecting first trial lens parameters for orthokeratology. This study aiming to compare four LLMs ChatGPT-4.1, ChatGPT-4o, ChatGPT-o3, and Claude 3.7 sonnet) in terms of subjective and objective evaluation to diagnosis of refractive errors, CRT lens fitting and selection of first trial lens parameters (Figure 2). The clinical and experimental data for this study were collected between June and September 2025.

Based on an expected effect size (*f* = 0.25), α = 0.05, β = 0.20, a minimum of 24 participants was calculated using G*Power for 80% power, this study included 30 patients who had been continuously wearing Paragon CRT 100 lenses (Paragon Vision Sciences, Gilbert, AZ, United States) at the Optometry Center of Xi’an People’s Hospital (Xi’an Fourth Hospital) since 2020. The present study was conducted in accordance with the principles of the Declaration of Helsinki, and ethical approval was obtained from the Ethics Committee of Xi’an People’s Hospital (Xi’an Fourth Hospital, approval number: 20220030).

Inclusion criteria were: (1) patients who underwent three to four lens replacements during fitting without switching brands and without significant ocular complications; (2) relatively complete clinical records including personal details, baseline ocular biometric parameters, the first trial lens and final prescription; and (3) both the first trial lens and final prescriptions determined by the same doctor to reduce inter-doctor variability. Exclusion criteria included incomplete fitting records, brand/design switching, discontinuation during fitting, clinically significant adverse events, or documented corneal conditions likely to confound ortho-k fitting.

### 2.2 Materials

All subjects were fitted with the Paragon CRT^®^ 100 lens (Paragon Vision Sciences, Gilbert, AZ, United States), composed of Paragon HDS 100 (pafluocon D) material and with an oxygen permeability DK/t of 140. The lenses have three zones: The central spherical zone with the base curve (BC), a mathematically designed sigmoidal corneal proximity “Return Zone” (RZD) and a non-curving “Landing Zone” (LZA). When fitting CRT lens, the first step is to select the first trial lens based on these parameters under manufacturer-defined constraints. Following the manufacturer-based fitting logic, the target BC power (mm) can be estimated as:


$$B\,C=\frac{337.5}{F\,K–M\,R\,S–0.50\,A\,d\,j\,u\,s\,t\,m\,e\,n\,t}$$

*FK:* flat Keratometric value

*MRS:* Manifest Refraction Sphere

*0.50 Adjustment*: the Jessen factor.

**FIGURE 1:**
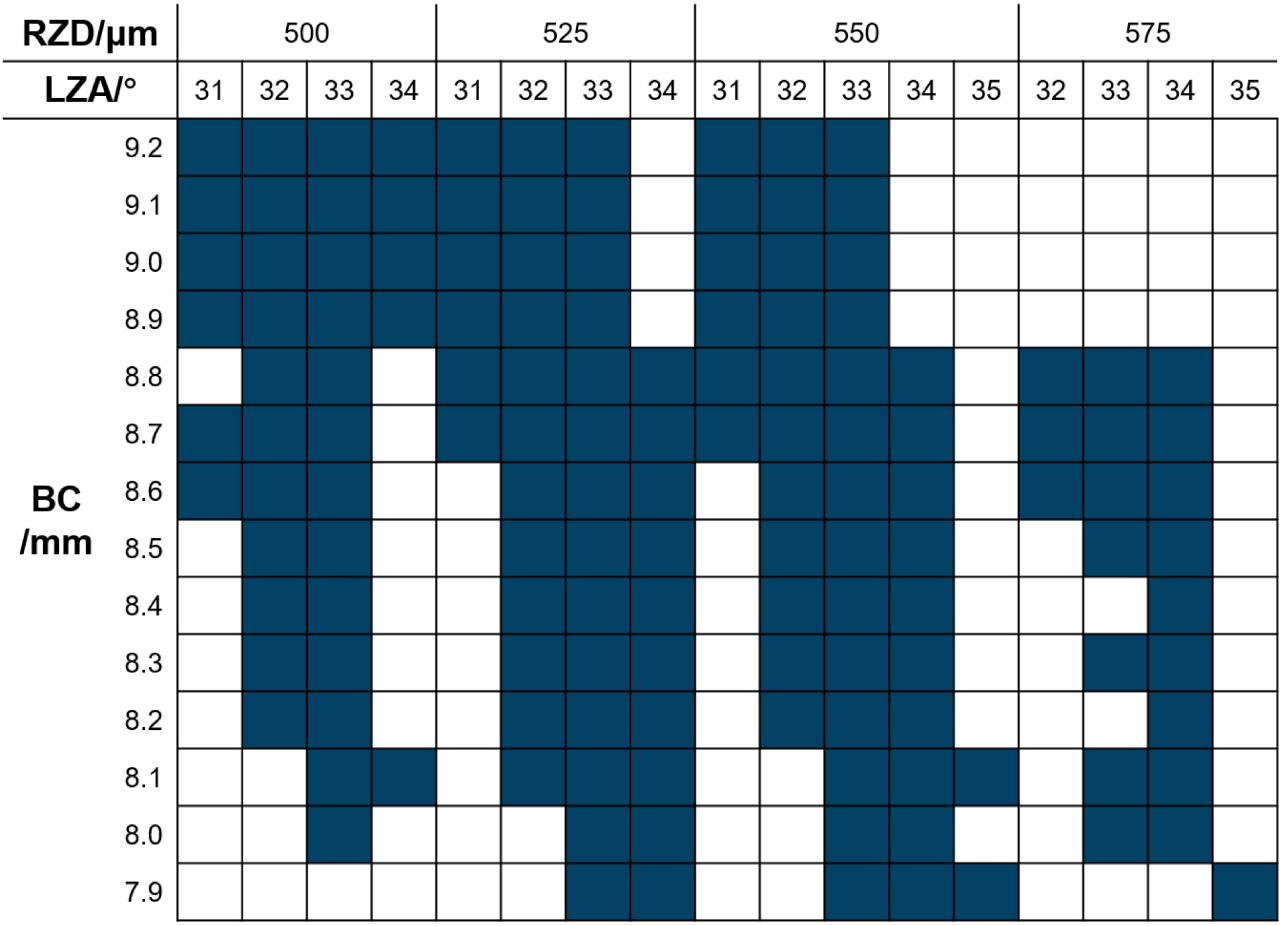
CRT trial lens selection diagram. Dark blue squares indicate available parameter combinations in the trial lens set. BC, base curve; RZD, return zone depth; LZA, landing zone angle.

RZD and LZA were selected according to the manufacturer’s fitting guide and trial lens availability constraints, and confirmed by clinicians based on fitting safety and feasibility. The CRT trial lens set contains 136 trial lenses, providing a wide range of parameter combinations to meet diverse clinical needs. The RZD options include 500–575 μm in 25 μm increments, the LZA options range from 31 to 35° in 1° steps, and the BC options range from 7.90 to 9.20 mm in 0.10 mm increments. The parameter combinations (dark blue squares) that can be used for selecting the first trial lens are shown in Figure 1. In this study, the total diameter (TD) was 10.5 mm, the back optic zone diameter was 6.0 mm, and no dual axis designs were used.

The Sirius corneal topography system (CSO, Italy) and the Medmont E300 (Medmont Pty Ltd., Melbourne, Vic, Australia) were used to obtain the corneal parameters, including the horizontal visible iris diameter (HVID), flat keratometric (K) reading (Flat K), steep K reading (Steep K), mean K reading (Mean K), e-value, ACD and the sag differential at 8 mm (SD8). An IOL Master (Carl Zeiss, Ltd., Germany) was used to measure the AL of all subjects. Cycloplegic refractions were examined 35 min after three instillations of 1% cyclopentolate, administered at 5-min intervals, and spherical and astigmatic refraction readings were obtained by an autorefractor. The basic clinical information of the subjects is shown in Table 1.

**TABLE 1:** Basic information of subjects and ocular parameters.

Characters	Mean ± SD	Range
Age (A)/(years)	9.5 ± 1.8	8.0 ∼ 16.0
Spherical refraction (S)/(D)*	−2.31 ± 0.79	−3.50 ∼−0.75
Cylindrical refraction (C)/(DC)*	−0.31 ± 0.35	−1.25 ∼−0.00
Axial length (AL)/(mm)	24.55 ± 0.59	23.28 ∼25.50
Anterior chamber depth (ACD)/(mm)	3.39 ± 0.23	2.96 ∼ 3.86
Horizontal visible iris diameter (HVID)/(mm)	11.87 ± 0.28	11.30 ∼ 12.71
Flat keratometric (FK)/(D)	42.60 ± 1.06	40.55 ∼ 45.14
Steep keratometric (SK)/(D)	43.60 ± 1.11	41.50 ∼ 46.35
Eccentricity value at 6 mm zone (E6)	0.48 ± 0.11	0.10 ∼ 0.76
Eccentricity value at 8 mm zone (E8)	0.57 ± 0.08	0.33 ∼ 0.74
Sag differential at 8 mm (SD8)/(μm)	19.00 ± 7.90	5.00 ∼ 41.00

*N* = 30; gender (G) male 15 (50.0%), female 15 (50.0%).

* These data were based on cycloplegic refraction.

### 2.3 LLM query protocol

**FIGURE 2:**
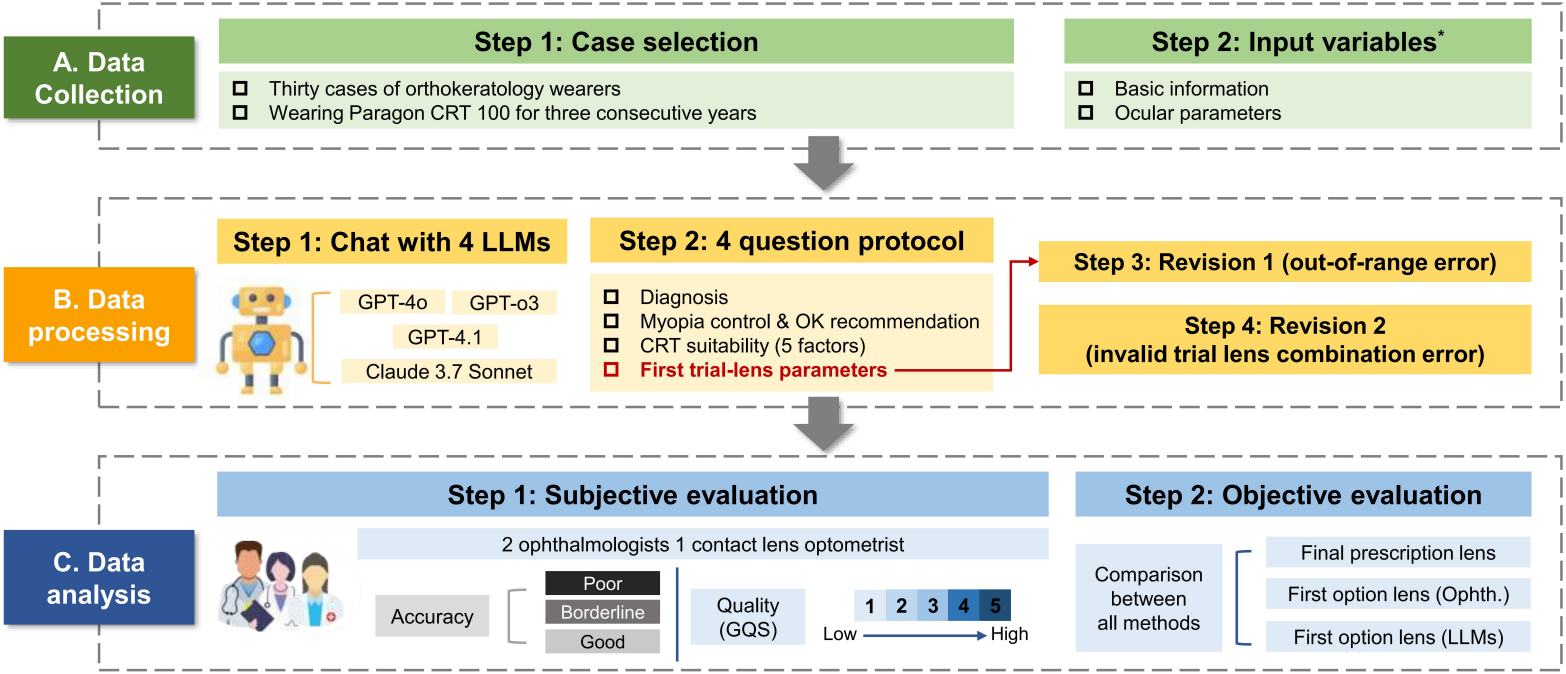
Overview of the methodological workflow. *Detailed information is available in Supplementary material.

We extracted each patient’s basic information and ocular parameters from clinical records. We organized them in a standardized input format (see Supplementary material for the complete input template). Following the clinical workflow of diagnosis and management, we designed a four-question query protocol (Figures 2 A, B). All questions and the query format were reviewed by all authors and finalized by consensus. Identical inputs and prompts were used across all LLMs. After each answer to a query, the memory was cleared for all LLMs to prevent the previous queries from influencing the subsequent outputs. Response contents were recorded in the table.

Q1. What is the most likely diagnosis?

Q2. Does this patient need myopia prevention and control? Are orthokeratology lenses recommended for prevention and control?

Q3. I would like to prefer Paragon CRT, please evaluate the patient’s fitness by giving a diagnosis from the following 5 points: Corneal eccentricity, Astigmatism, Corneal diameter, Corneal curvature, Lens customization.

Q4. Please give the parameters of the first trial lens of Paragon CRT.

To assess the feasibility of the trial lens parameters, a predefined two-step correction protocol (a maximum of 2 rounds) was applied only to Q4. First, the initial Q4 output was checked for clearly out-of-range values (e.g., implausible RZD values outside the product design range). If present, we initiate the first revision “Some parameters are incorrect, please modify.” Second, we verified whether the predicted parameter combination was available within the manufacturer’s CRT trial lens selection set (Figure 1). If the combination was within the custom lens range but not available as a trial-lens option, the second round of revisions commences: “Please refer to the CRT trial lens selection for the correct parameter combination.” Importantly, these correction prompts did not disclose any ground-truth final prescription values; they only imposed feasibility constraints (product design range and trial-lens availability).

The correction procedure was capped at two rounds to avoid open-ended optimization and to reflect a minimal, practical clinical interaction. The complete query templates (including correction prompts and formatting requirements) are provided in Supplementary material.

### 2.4 Research workflow

The general research workflow is illustrated in Figure 2. Briefly, cases were collected according to the predefined inclusion/exclusion criteria and converted into a standardized case template for LLM queries. Each case was then processed using a four-question protocol across four LLMs (GPT-4o, GPT-o3, GPT-4.1, and Claude 3.7 Sonnet), with the two-round correction applied only to the trial-lens parameter task (Q4) to ensure feasibility. Specifically, Revision 1 addressed out-of-range errors, defined as any predicted trial lens parameter (BC/RZD/LZA/TD) falling outside the manufacturer-specified Paragon CRT product design range (parameter-level counting). Revision 2 addressed invalid trial lens combination errors, defined as outputs in which parameter values were individually within the customizable range. However, the parameter combination did not correspond to an available option in the manufacturer’s CRT trial lens selection set (at the combination level). Finally, the evaluation of the LLMs performance involved both subjective and objective analysis. Subjective evaluation applies two assessments: one uses the global quality score (GQS) to evaluate overall quality, while the other applies a three-tier grading system to evaluate accuracy. The evaluation was conducted by two ophthalmologists and one contact lens optometrist (WJH, WJQ, and LSG) who are highly professional and have over 5 years of clinical and education experience. The identities of the LLMs were masked from the graders to maintain objectivity. The objective evaluation entailed comparing the parameters of the initial trial lenses recommended by each LLM against the final prescription determined by the ophthalmologists.

### 2.5 Grading criteria of subjective evaluation

All responses generated by each LLM are evaluated across two dimensions: overall quality and accuracy. Overall quality is assessed using the Global Quality Score (GQS), which consists of a 5-point Likert scale with scores ranging from 1 (poor quality) to 5 (excellent quality). The accuracy scoring criteria comprise three distinct levels: “poor” for significant inaccuracies with potential to harm, “borderline” for minor factual errors unlikely to mislead, and “good” for fully accurate and clinically appropriate recommendations. The scoring process is conducted independently according to the above definitions, with the final score determined by averaging the ratings from three experts and performing inter-rater reliability analysis. Inter-rater agreement was quantified using the Intraclass Correlation Coefficient (ICC) based on a two-way mixed-effects model with absolute agreement. We report both single-measure reliability [ICC (3,1)] and average-measure reliability for the mean of three raters [ICC (3,3)]. For the three-level accuracy ratings, categories were coded as ordinal scores (1–3) to enable ICC-based reliability estimation.

### 2.6 Statistical analysis

In this study, continuous variables were presented as mean ± standard deviation (SD) for descriptive purposes and as median with interquartile range (IQR) to align with the non-parametric statistical approach. Categorical variables were summarized by frequency and percentage. The parameter differences between the initial trial lenses (ophthalmologists and four LLMs) and the final prescription were compared using the Wilcoxon signed-rank test for paired continuous data. Agreement between methods was further evaluated using Bland–Altman plots, reporting the mean bias and 95% limits of agreement (mean ± 1.96 SD). Friedman’s test was applied to detect overall differences among the four models of Global Quality Score (GQS) ratings, followed by pairwise Wilcoxon signed-rank tests with Bonferroni correction for multiple comparisons. Applying the Kruskal-Wallis test to compare the first trial lens parameters difference among the four methods. All data were analyzed using the IBM SPSS Statistics for Windows (SPSS, ver. 25.0), and figures were generated using MATLAB (R2018b; MathWorks, Natick, MA, United States). In all cases, *p* < 0.05 was considered statistically significant.

## 3 Results

### 3.1 Overall quality and accuracy

**FIGURE 3:**
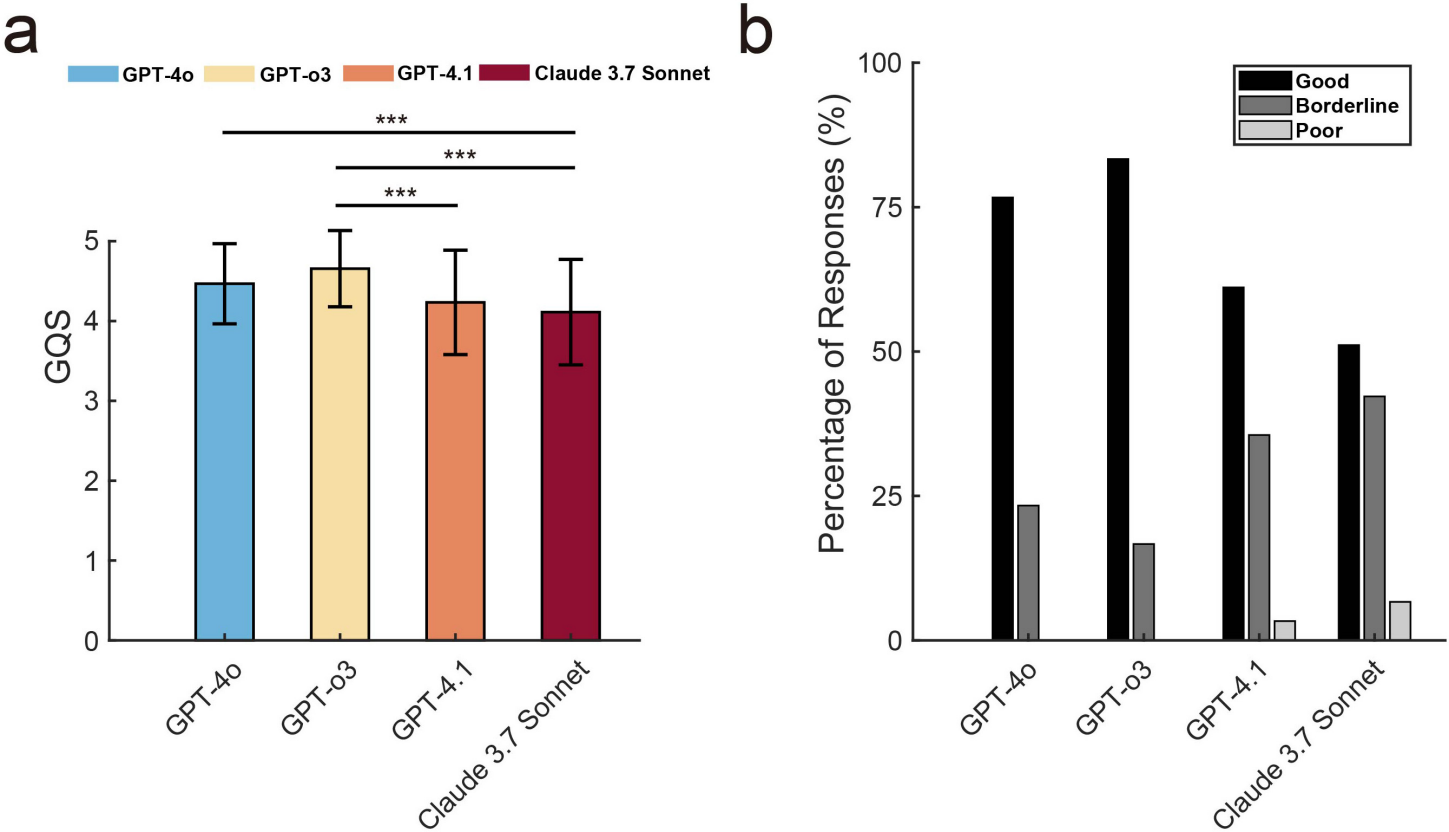
The global quality score (GQS) and the accuracy assessment for text generated by large language models. **(a)** GQS comparison across four LLMs (mean ± SD). Three asterisks (***) stand for *p* < 0.001. **(b)** Distribution of three-level accuracy ratings (Good/Borderline/Poor) across LLMs, shown as percentages of evaluated responses.

The Friedman test revealed a statistically significant difference in GQS among the four LLMs [χ^2^(3) = 39.85, *p* < 0.001, Kendall’s *W* = 0.148], suggesting variability in response quality across the LLMs (Figure 3a). *Post hoc* Wilcoxon signed-rank tests with Bonferroni correction (adjusted α = 0.0083) demonstrated that the GQS of ChatGPT-o3 (4.66 ± 0.48) was significantly higher than the GPT-4.1 score (4.23 ± 0.65, *p* < 0.001) and the Claude 3.7 Sonnet score (4.11 ± 0.66, *p* < 0.001). Similarly, ChatGPT-4o (4.47 ± 0.5) also outperformed Claude 3.7 Sonnet (*p* < 0.001). The Intraclass Correlation Coefficient (ICC) for the GQS scores was 0.633 (*p* < 0.001; 95% CI: 0.543–0.715) for ICC (3,1) and ICC (3,3) was 0.838 (*p* < 0.001; 95% CI: 0.781–0.883), indicating a relatively high level of consistency across raters when averaging the scores.

Figure 3b illustrates the three-tiered analysis of response accuracy across four language models. GPT-3 achieved the highest percentage of “good” responses (83.3%), followed by GPT-4o (76.7%) and GPT-4.1 (61.1%). Meanwhile, Claude 3.7 Sonnet recorded a comparatively low percentage (51.1%). Regarding “borderline” responses, GPT-4o recorded 23.3%, GPT-o3 16.7%, GPT-4.1 35.6%, and Claude 3.7 Sonnet 42.2%. Notably, neither GPT-4o nor GPT-o3 produced responses rated as “poor,” whereas GPT-4.1 and Claude 3.7 Sonnet exhibited 3.3% and 6.7% such instances, respectively. The ICC for accuracy assessments among the three raters indicates good consistency and stability, as evidenced by 0.459 (*p* < 0.001; 95% CI: 0.350–0.564) for ICC (3,1) and ICC (3,3) was 0.718 (*p* < 0.001; 95% CI: 0.618–0.795).

### 3.2 Effect of two-step correction on output errors

**FIGURE 4:**
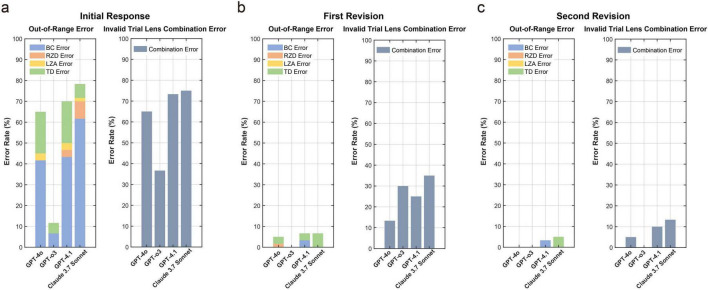
Out-of-range and invalid trial lens combination error rates for first trial-lens parameters across **(a)** the initial response **(b)** first revision **(c)** second revision for the four LLMs. No histogram columns indicate a ratio of 0.

Figure 4 summarizes error rates of first trial-lens parameter outputs across the initial response and two revision rounds. For out-of-range errors, the initial response vs. first revision rates were GPT-4o 65% vs. 5%, GPT-o3 37% vs. 0%, GPT-4.1 73% vs. 7%, and Claude 3.7 Sonnet 75% vs. 7%. At the initial stage, BC accounted for the most frequent out-of-range parameter with BC out-of-range rates of 42% (GPT-4o), 7% (GPT-o3), 43% (GPT-4.1), and 62% (Claude 3.7 Sonnet). After the first revision, BC out-of-range errors were only in GPT-4.1, showing residual BC out-of-range errors (3%), and the other models showed 0% for BC errors. For invalid trial-lens combination errors, the first revision and second revision (Figure 3b vs. Figure 4c) rates were GPT-4o 13% vs. 5%, GPT-o3 30% vs. 0%, GPT-4.1 25% vs. 10%, and Claude 3.7 Sonnet 35% vs. 13%. After the second revision, only GPT-o3 achieved 0% invalid-combination errors, whereas the other models still had residual errors.

### 3.3 Final prescription vs. first option in parameters distribution and adjustment step

Table 2 presents a comparison between an ophthalmologist and four LLMs (GPT-4o, GPT-o3, GPT-4.1, and Claude 3.7 Sonnet) in the selection of parameters for the first trial lens and final prescription, as tested with the Wilcoxon signed-rank test. The results showed the ophthalmologist’s trial lens differed significantly from the final prescription only in BC (*Z* = –2.95, *p* = 0.003), while no significant differences were observed in RZD (*Z* = –1.26, *p* = 0.21) or LZA (*Z* = –0.90, *p* = 0.37). In contrast, all LLMs demonstrated statistically significant differences from the final prescription in these parameters, with GPT-4o (*Z* = –4.21, *p* < 0.001), GPT-o3 (*Z* = –3.78, *p* < 0.001), GPT-4.1 (*Z* = –4.85, *p* < 0.001), and Claude 3.7 Sonnet (*Z* = –3.96, *p* < 0.001). DIA showed a statistical difference (*Z* = –2.03, *p* = 0.041), but the lens diameter was fixed at 10.5 mm, and was excluded from further analyses.

**TABLE 2:** Comparison of actual average parameters: final prescription lenses vs. first option lenses of ophthalmologist and four LLMs.

Group		BCR (mm)		RZD (μm)		LZA (°)		TD (mm)	
		Mean ± SD	Median [IQR]	Mean ± SD	Median [IQR]	Mean ± SD	Median [IQR]	Mean ± SD	Median [IQR]
Final prescription		8.7 ± 0.3	8.8 [8.5–8.9]	528.8 ± 17.1	525.0 [525.0–531.2]	32.6 ± 1.2	33.0 [32.0–33.0]	10.6 ± 0.2	10.5 [10.5–11.0]
First option	Ophth.	8.6 ± 0.3	8.6 [8.4–8.8]***	527.1 ± 13.3	525.0 [525.0–525.0]	32.7 ± 0.6	33.0 [32.0–33.0]	10.5 ± 0.0	10.5 [10.5–10.5]***
	GPT-4o	8.3 ± 0.3	8.3 [8.0–8.5]***	545.2 ± 11.0	550.0 [550.0–550.0]***	33.1 ± 0.3	33.0 [33.0–33.0]***		
	GPT-o3	8.4 ± 0.3	8.4 [8.1–8.6]***	548.8 ± 7.2	550.0 [550.0–550.0]***	33.3 ± 0.5	33.0 [33.0–34.0]***		
	GPT-4.1	8.1 ± 0.2	8.0 [7.9–8.3]***	548.7 ± 10.0	550.0 [550.0–550.0]***	33.3 ± 0.4	33.0 [33.0–34.0]***		
	Claude 3.7 Sonnet	8.4 ± 0.3	8.4 [8.1–8.6]***	543.3 ± 17.9	550.0 [525.0–550.0]***	32.9 ± 0.7	33.0 [33.0–33.0]*		

Mean ± SD are presented for descriptive purposes; median [IQR] are provided for consistency with the Wilcoxon signed-rank test.

*indicated *p-*value < 0.05;

***indicated *p*-value ≤ 0.001. BCR, base curve radius; RZD, return zone depth; LZA, landing zone angle; TD, total lens diameter; Ophth., ophthalmologist.

To further evaluate the differences among the five ways, Kruskal–Wallis test revealing significant group differences in BC (χ^2^ = 45.2, *p* < 0.001), RZD (χ^2^ = 32.7, *p* < 0.001), and LZA (χ^2^ = 27.8, *p* < 0.001). *Post hoc* pairwise comparisons were conducted with Bonferroni correction (adjusted α = 0.0083). The ophthalmologist differed significantly from all LLMs in BC (all *p*s < 0.0083), and GPT-4.1 produced significantly lower BC values than the other LLMs (*p* < 0.001). For RZD, all LLMs yielded consistently higher values compared with the ophthalmologist (*p* < 0.001), while no significant differences were observed among them (all *p*s > 0.0083). Regarding LZA, Claude 3.7 Sonnet was significantly lower than GPT-o3 (*p* = 0.004) and GPT-4.1 (*p* = 0.002), but was comparable to GPT-4o (*p* = 0.12) and the ophthalmologist (*p* = 0.18).

**FIGURE 5:**
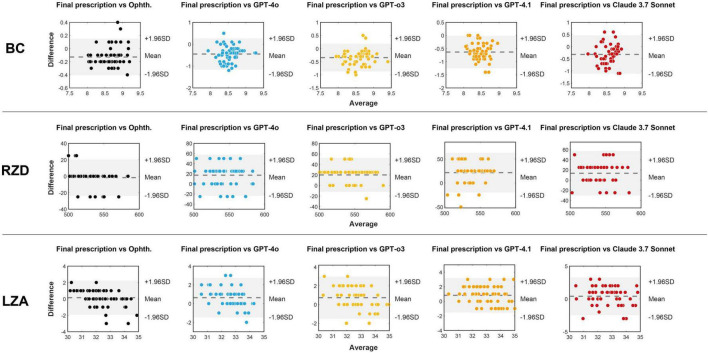
Bland-Altman plots between the three parameters (BC, RZD, and LZA) of the final prescribed lens and the first option lenses provided by the ophthalmologist and four LLMs. The *x*-axis represents the average of every method for first trial lens and the final prescribed lens parameters, and the *y*-axis represents the difference between them. The dashed line indicates the mean bias, while the shaded area corresponds to the 95% limits of agreement (mean bias ± 1.96 SD). Ophth., ophthalmologist.

Bland–Altman analyses were further performed to evaluate the consistency between the five methods (ophthalmologist and four LLMs) for selecting the first trial lens and the final prescribed lens parameters. As shown in Figure 5, most data points fell within the 95% limits of agreement across methods. The plots also revealed systematic tendencies in the LLM-generated parameters, including BC underestimation and RZD overestimation.

**FIGURE 6:**
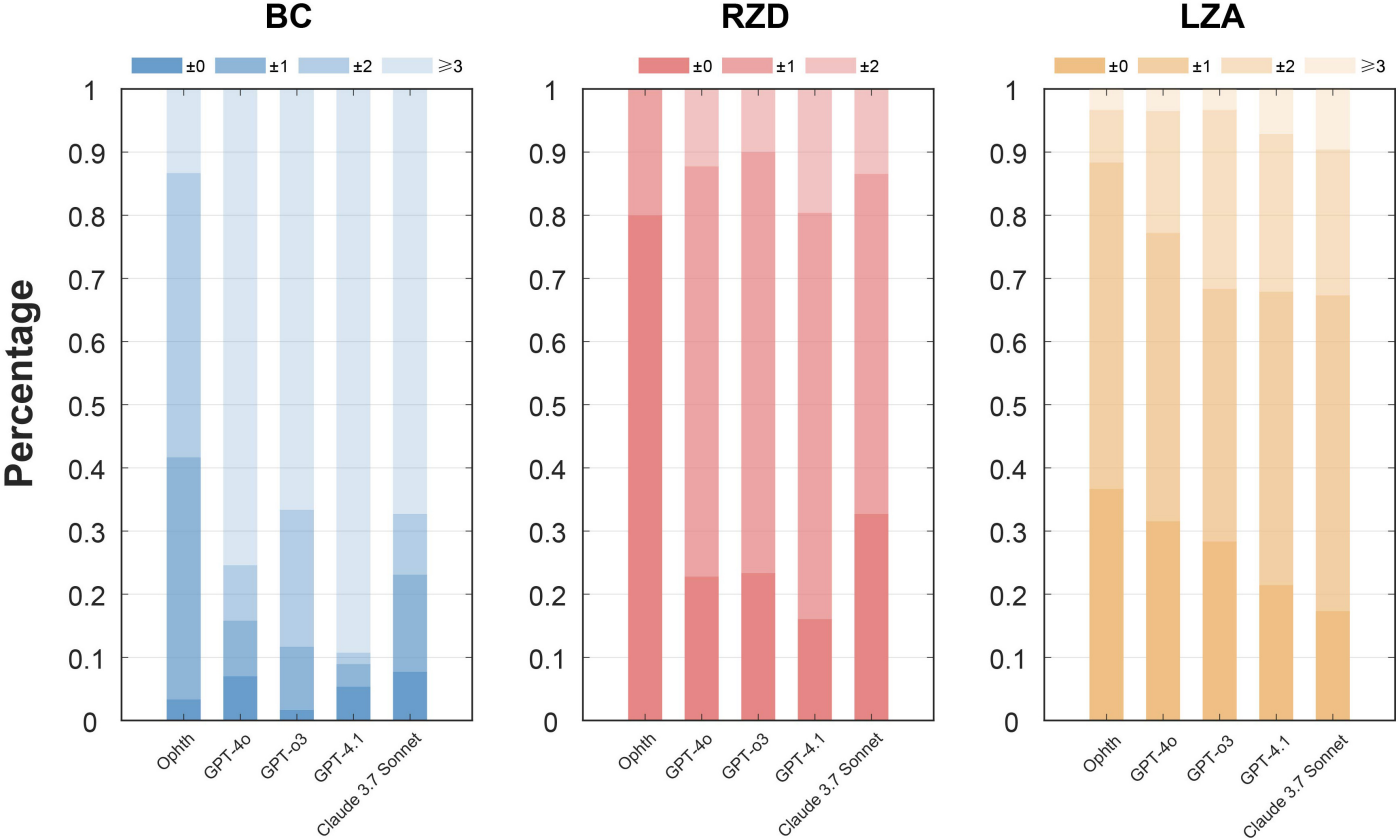
Step deviation levels for parameters BCR, RZD, and LZA between the first trial lenses and the final prescription. The figure illustrates the percentage of deviations categorized into different levels, with the darker color bars indicating more minor adjustments and the lighter bars indicating larger deviations. The *x*-axis represents different ways, and the *y*-axis represents the deviation level rate.

Table 3 and Figure 6 detail the deviations in BCR, RZD, and LZA between the first trial lens provided in different ways and the final prescription. For BC, which showed the most significant deviation among all parameters, the ophthalmologist’s trial lenses required adjustments within two steps in 86.7% of cases. In contrast, LLMs showed much lower percentages, with GPT-4.1 achieving only 10.7%. All LLMs consistently gave smaller BC values, with GPT-4.1 presenting the most significant deviation. Regarding RZD, all LLMs slightly overestimated the values; however, in more than 80% of the cases, no adjustment or only a one-step adjustment was required. Specifically, GPT-4o (87.7%), GPT-o3 (90%), GPT-4.1 (80.4%), and Claude 3.7 Sonnet (86.5%) all performed reasonably well. Finally, both the ophthalmologist and the language models demonstrated good agreement regarding LZA, with over 95% of cases falling within two steps of adjustment for the ophthalmologist, GPT-4o, and GPT-o3. In contrast, GPT-4.1 (92.9%) and Claude 3.7 Sonnet (90.4%) showed slightly lower consistency. In summary, LLMs performed poorly in BC, with GPT-4.1 showing the lowest accuracy on selecting parameters.

**TABLE 3:** Deviations of three parameters (BC, RZD, and LZA) between final prescription lenses and first option lenses (ophthalmologist and four LLMs).

Group	BCR (mm)	RZD (μm)	LZA (°)
Ophth.	8.6 ± 2 Step	525.0 ± 1 Step	33.0 ± 2 Step
GPT-4o	8.3 ± 4 Step	550.0 ± 1 Step	33.0 ± 2 Step
GPT-o3	8.4 ± 3 Step	550.0 ± 1 Step	33.0 ± 2 Step
GPT-4.1	8.1 ± 4 Step	550.0 ± 1 Step	33.0 ± 2 Step
Claude 3.7 sonnet	8.4 ± 5 Step	550.0 ± 1 Step	33.0 ± 2 Step

Values are expressed as mean ± SD. 1 step is 0.10 mm for BCR, 25 μm for RZD and 1° for LZA. BCR, base curve radius; RZD, return zone depth; LZA, landing zone angle; Ophth., ophthalmologists.

## 4 Discussion

This study evaluated LLMs as assistive tools for orthokeratology fitting workflow that includes diagnostic reasoning, CRT suitability assessment, and first trial lens parameter suggestion. We found that when manufacturer feasibility constraints were not explicitly enforced, parameter outputs contained more out-of-range values and invalid trial-lens combinations. After applying a predefined two-round correction protocol, error rates decreasing across models and the GPT series models have lower error rate. When subjective evaluated on the complete response, GPT-4o and GPT-o3 achieving higher GQS scores and accuracy assessment. In the first trial lens parameter analysis, differences between LLMs and clinicians were mainly concentrated in BC and RZD, with a directional tendency toward a smaller BC radius (mm) and a larger RZD. Collectively, these findings support the view that LLMs may be a promising decision-support tool under a constraint-guided workflow, while underscoring the need for clinician oversight and verification in trial-lens parameter selection.

We observed some biases in LLMs-generated first trial lens parameters, most notably a tendency toward a smaller BC radius (mm) and a larger RZD than in clinician-derived parameters. These biases are clinically meaningful because BC and RZD jointly influence sagittal depth and the fluorescein pattern, and systematic deviations can increase the number of trial lens exchanges required to reach the final prescription. For the BC values of CRT trial lenses, the calculation method is the flat K value minus the absolute value of the target refractive error and the Jessen factor (fixed at +0.50). In routine clinical practice, clinicians often do not apply this rule mechanically. Instead, they may intentionally incorporate additional treatment targets or overcorrection beyond the manifest refraction, and adjust the effective Jessen factor based on patient-specific considerations and evidence on myopia control (35). Such expert adjustments can yield a flatter trial lens choice, corresponding to a larger BC radius. In contrast, LLMs output frequently adhered rigidly to the manifest refractive error and a fixed Jessen factor (+ 0.50 D), which can produce a steeper BC estimate and thus a smaller BC radius (mm) relative to clinician choices. This difference in clinical strategy provides a plausible explanation for the observed systematic underestimation of BC. In addition to differences in clinical strategy, we identified inconsistent or incorrect rule application in some LLM responses. For example, some outputs omitted the refractive term during the adjustment step, used flat K directly without incorporating a treatment target, or applied non-manufacturer-specific additive adjustments to keratometry before converting to millimeters. These inconsistencies, inferred from the LLMs’ stated calculation steps and the resulting parameter values, likely contribute to variability and amplify BC deviations.

RZD selection is typically guided by manufacturer recommendations and refined by the observed fitting pattern. Clinicians commonly adopt a conservative approach when choosing RZD to balance treatment effect with safety and stability, particularly in borderline situations. This safety-oriented heuristic often favors shallower RZD selections, whereas LLMs may default to deeper values or fail to integrate conservative decision rules consistently. Given that RZD is discretized in fixed increments, even a one-step shift can result in systematic overestimation. Our findings of larger RZD outputs are therefore consistent with the notion that LLMs can generate numerically plausible values while underweighting expert priors that prioritize conservative fitting and safety.

In this study, LLMs provided generally high quality and accurate responses for the CRT-related workflow when evaluated on the complete response, which is relatively superior compared to other ophthalmological studies (30, 36). This may be attributed to the fact that the patient cases selected for this study were highly typical, as these patients had already undergone rigorous screening before receiving corneal reshaping lenses. However, we observed that feasibility errors (out-of-range values and invalid trial-lens combinations) were more common when constraints were not explicitly enforced, and they decreased after applying the predefined correction workflow, indicating that constraint guidance can improve the practical usability of LLM outputs for subsequent analyses. Collectively, these findings suggest that LLMs may be useful as assistive decision-support tools within a constraint-guided workflow, while underscoring that clinician oversight remains necessary.

The increasing prevalence and earlier onset of myopia have amplified clinical workload and motivated scalable decision support tools in optometry. Artificial intelligence has shown substantial potential in ophthalmology, particularly for assisting clinical assessment, screening, and diagnostic decision making. Deep learning methods applied to ocular imaging have been used for pediatric screening of myopia, strabismus, amblyopia, and fundus related abnormalities, where efficiency and scalability are important for real world practice (37–40). Regarding interventions, especially orthokeratology, recent studies have increasingly used machine learning approaches to optimize first trial lens selection and to predict corneal reshaping characteristics after wear, such as treatment zone changes and eccentricity (41–45). However, orthokeratology fitting remains highly individualized, and post-trial adjustments depend on factors beyond baseline parameters (e.g., eyelid tension, tear-film stability, lens centration, and fluorescein-pattern interpretation). Compared with numeric tools that only provide direct input and output, an interactive workflow that supports conversation and iterative clarification may better assist adjustment planning, provided that outputs are used under manufacturer feasibility constraints and clinician oversight, given the risk of inconsistency and error in LLM generated suggestions.

Our study also has some limitations. First, our dataset mainly consisted of clinically typical CRT candidates, which may inflate apparent performance and limits generalizability to more challenging presentations (e.g., higher astigmatism, atypical corneal morphology, or failed trial fittings). Future prospective studies should intentionally enrich borderline and challenging subgroups and perform stratified analyses. Second, we focused on a single lens design (Paragon CRT); extending evaluation to additional mainstream orthokeratology designs will be important, but will require design-specific protocols and constraints. Third, this study focused on first trial-lens parameter suggestion and did not evaluate downstream fitting dynamics, repeated follow-up adjustments, or long-term outcomes. Although some LLM responses included post-trial adjustment suggestions, the safety and accuracy of such recommendations require dedicated validation before clinical use. Although our analyses focused on first trial lens parameter suggestion, we noted that some LLM responses also described potential post-trial fitting outcomes and adjustment considerations; future work could build on this by conducting prospective studies to test whether LLM assisted guidance improves fitting efficiency or decision consistency for less experienced clinicians under clinician supervision.

## 5 Conclusion

In summary, this study found that language models have the potential to analyze patients’ ocular parameters and provide the first trial lens. Overall, GPT-o3 and GPT-4o demonstrated the strongest performance in response quality and accuracy when evaluated on the complete response. For first trial lens parameter generation, outputs frequently required clinician supervision through feasibility constraints, and relative to the first and final prescription by clinician, discrepancies were mainly concentrated in key fitting parameters, particularly BC and RZD, showing a directional tendency toward BC underestimation and RZD overestimation. These findings suggest that LLMs may serve as assistive decision-support tools for routine CRT-related workflows when combined with manufacturer constraints and clinician verification.

## Data Availability

The raw data supporting the conclusions of this article will be made available by the authors, without undue reservation.

## References

[1] Holden BA Fricke TR Wilson DA Jong M Naidoo KS Sankaridurg P Global prevalence of myopia and high myopia and temporal trends from 2000 through 2050. *Ophthalmology*. (2016) 123:1036–42. 10.1016/j.ophtha.2016.01.006 26875007

[2] Khanal S Tomiyama ES Harrington SC. Childhood myopia part i: contemporary treatment options. *Invest Ophthalmol Vis Sci*. (2025) 66:6. 10.1167/iovs.66.7.6 40471570 PMC12151248

[3] Zhang XJ Zaabaar E French AN Tang FY Kam KW Tham CC Advances in myopia control strategies for children. *Br J Ophthalmol*. (2025) 109:165–76. 10.1136/bjo-2023-323887 38777389

[4] González-Méijome JM Villa-Collar C. Nomogram, corneal topography, and final prescription relations for corneal refractive therapy. *Optom Vis Sci*. (2007) 84:59–64. 10.1097/01.opx.0000254633.32449.89 17220779

[5] Sánchez-García A Molina-Martin A Ariza-Gracia MÁ Piñero DP. Analysis of treatment discontinuation in orthokeratology: studying efficacy, safety, and patient adherence over six months. *Eye Contact Lens.* (2024) 50:395–400. 10.1097/ICL.0000000000001110 38886923

[6] Sun Y Peng Z Zhao B Hong J Ma N Li Y Comparison of trial lens and computer-aided fitting in orthokeratology: a multi-center, randomized, examiner-masked, controlled study. *Cont Lens Anterior Eye*. (2024) 47:102172. 10.1016/j.clae.2024.102172 38806329

[7] Nichols JJ Jones L Morgan PB Efron N. Bibliometric analysis of the orthokeratology literature. *Cont Lens Anterior Eye*. (2021) 44:101390. 10.1016/j.clae.2020.11.010 33298369

[8] Zhang Z Zhou J Zeng L Xue F Zhou X Chen Z. The effect of corneal power distribution on axial elongation in children using three different orthokeratology lens designs. *Cont Lens Anterior Eye*. (2023) 46:101749. 10.1016/j.clae.2022.101749 36008212

[9] Bullimore MA Johnson LA. Overnight orthokeratology. *Cont Lens Anterior Eye*. (2020) 43:322–32. 10.1016/j.clae.2020.03.018 32331970

[10] Gormley DJ Gersten M Koplin RS Lubkin V. Corneal modeling. *Cornea.* (1988) 7:30–5. 10.1097/00003226-198801000-00004 3349789

[11] Wang J Fonn D Simpson TL Sorbara L Kort R Jones L. Topographical thickness of the epithelium and total cornea after overnight wear of reverse-geometry rigid contact lenses for myopia reduction. *Invest Ophthalmol Vis Sci*. (2003) 44:4742–6. 10.1167/iovs.03-0239 14578394

[12] Vincent SJ Cho P Chan KY Fadel D Ghorbani-Mojarrad N González-Méijome JM CLEAR - Orthokeratology. *Cont Lens Anterior Eye*. (2021) 44:240–69. 10.1016/j.clae.2021.02.003 33775379

[13] Thirunavukarasu AJ Ting DSJ Elangovan K Gutierrez L Tan TF Ting DSW. Large language models in medicine. *Nat Med*. (2023) 29:1930–40. 10.1038/s41591-023-02448-8 37460753

[14] Bedi S Liu Y Orr-Ewing L Dash D Koyejo S Callahan A Testing and evaluation of health care applications of large language models: a systematic review. *JAMA*. (2025) 333:319–28. 10.1001/jama.2024.21700 39405325 PMC11480901

[15] Kung TH Cheatham M Medenilla A Sillos C De Leon L Elepaño C Performance of ChatGPT on USMLE: potential for AI-assisted medical education using large language models. *PLoS Digit Health*. (2023) 2:e0000198. 10.1371/journal.pdig.0000198 36812645 PMC9931230

[16] Ali R Tang OY Connolly ID Zadnik Sullivan PL Shin JH Fridley JS Performance of ChatGPT and GPT-4 on neurosurgery written board examinations. *Neurosurgery.* (2023) 93:1353–65. 10.1227/neu.0000000000002632 37581444

[17] Fraser H Crossland D Bacher I Ranney M Madsen T Hilliard R. Comparison of diagnostic and triage accuracy of ada health and WebMD symptom checkers, ChatGPT, and physicians for patients in an emergency department: clinical data analysis study. *JMIR Mhealth Uhealth*. (2023) 11:e49995. 10.2196/49995 37788063 PMC10582809

[18] Pagano S Holzapfel S Kappenschneider T Meyer M Maderbacher G Grifka J Arthrosis diagnosis and treatment recommendations in clinical practice: an exploratory investigation with the generative AI model GPT-4. *J Orthop Traumatol*. (2023) 24:61. 10.1186/s10195-023-00740-4 38015298 PMC10684473

[19] Cadamuro J Cabitza F Debeljak Z De Bruyne S Frans G Perez SM Potentials and pitfalls of ChatGPT and natural-language artificial intelligence models for the understanding of laboratory medicine test results. An assessment by the European Federation of Clinical Chemistry and Laboratory Medicine (EFLM) Working Group on Artificial Intelligence (WG-AI). *Clin Chem Lab Med*. (2023) 61:1158–66. 10.1515/cclm-2023-0355 37083166

[20] Barash Y Klang E Konen E Sorin V. ChatGPT-4 assistance in optimizing emergency department radiology referrals and imaging selection. *J Am Coll Radiol*. (2023) 20:998–1003. 10.1016/j.jacr.2023.06.009 37423350

[21] Walton N Gracefo S Sutherland N Kozel BA Danford CJ McGrath SP. Evaluating chatgpt as an agent for providing genetic education. *bioRxiv [Preprint].* (2023). 10.1101/2023.10.25.564074 38076902 PMC10705538

[22] Aiumtrakul N Thongprayoon C Arayangkool C Vo KB Wannaphut C Suppadungsuk S Personalized medicine in Urolithiasis: ai chatbot-assisted dietary management of oxalate for kidney stone prevention. *J Pers Med*. (2024) 14:107. 10.3390/jpm14010107 38248809 PMC10817681

[23] Kassab J Hadi El Hajjar A Wardrop RM Brateanu A. Accuracy of online artificial intelligence models in primary care settings. *Am J Prev Med*. (2024) 66:1054–9. 10.1016/j.amepre.2024.02.006 38354991

[24] Dağci M Çam F Dost A. Reliability and quality of the nursing care planning texts generated by ChatGPT. *Nurse Educ*. (2024) 49:E109–14. 10.1097/NNE.0000000000001566 37994523

[25] Tan DNH Tham YC Koh V Loon SC Aquino MC Lun K Evaluating Chatbot responses to patient questions in the field of glaucoma. *Front Med*. (2024) 11:1359073. 10.3389/fmed.2024.1359073 39050528 PMC11267485

[26] Kang D Wu H Yuan L Shen W Feng J Zhan J Evaluating the efficacy of large language models in guiding treatment decisions for pediatric refractive error. *Ophthalmol Ther*. (2025) 14:705–16. 10.1007/s40123-025-01105-2 39985747 PMC11920547

[27] Huang Y Shi R Chen C Zhou X Zhou X Hong J Evaluation of large language models for providing educational information in orthokeratology care. *Cont Lens Anterior Eye*. (2025) 48:102384. 10.1016/j.clae.2025.102384 39939269

[28] Srinivasan S Ji H Chen DZ Wong W Soh ZD Goh JHL Can off-the-shelf visual large language models detect and diagnose ocular diseases from retinal photographs? *BMJ Open Ophthalmol*. (2025) 10:e002076. 10.1136/bmjophth-2024-002076 40194867 PMC11977474

[29] Su Z Jin K Wu H Luo Z Grzybowski A Ye J. Assessment of large language models in cataract care information provision: a quantitative comparison. *Ophthalmol Ther*. (2025) 14:103–16. 10.1007/s40123-024-01066-y 39516445 PMC11724831

[30] Lim ZW Pushpanathan K Yew SME Lai Y Sun CH Lam JSH Benchmarking large language models’ performances for myopia care: a comparative analysis of ChatGPT-3.5. ChatGPT-4.0, and Google Bard. *EBioMedicine*. (2023) 95:104770. 10.1016/j.ebiom.2023.104770 37625267 PMC10470220

[31] Huang AS Hirabayashi K Barna L Parikh D Pasquale LR. Assessment of a large language model’s responses to questions and cases about glaucoma and retina management. *JAMA Ophthalmol*. (2024) 142:371–5. 10.1001/jamaophthalmol.2023.6917 38386351 PMC10884943

[32] Betzler BK Chen H Cheng CY Lee CS Ning G Song SJ Large language models and their impact in ophthalmology. *Lancet Digit Health*. (2023) 5:e917–24. 10.1016/S2589-7500(23)00201-7 38000875 PMC11003328

[33] Li X Zhang Y Zheng T Deng Y Lu Y Hu J Using large language models to generate child-friendly education materials on myopia. *Digit Health*. (2025) 11:20552076251362338. 10.1177/20552076251362338 40755959 PMC12317229

[34] Delsoz M Hassan A Nabavi A Rahdar A Fowler B Kerr NC Large language models: pioneering new educational frontiers in childhood myopia. *Ophthalmol Ther*. (2025) 14:1281–95. 10.1007/s40123-025-01142-x 40257570 PMC12069199

[35] Lau JK Wan K Cho P. Orthokeratology lenses with increased compression factor (OKIC): a 2-year longitudinal clinical trial for myopia control. *Cont Lens Anterior Eye*. (2023) 46:101745. 10.1016/j.clae.2022.101745 35995721

[36] He HJ Zhao FF Liang JJ Wang Y He QQ Lin H Evaluation and comparison of large language models’ responses to questions related optic neuritis. *Front Med*. (2025) 12:1516442. 10.3389/fmed.2025.1516442 40636386 PMC12238082

[37] Yang Y Li R Lin D Zhang X Li W Wang J Automatic identification of myopia based on ocular appearance images using deep learning. *Ann Transl Med*. (2020) 8:705. 10.21037/atm.2019.12.39 32617325 PMC7327333

[38] Chen Z Fu H Lo WL Chi Z. Strabismus recognition using eye-tracking data and convolutional neural networks. *J Healthc Eng*. (2018) 2018:7692198. 10.1155/2018/7692198 29854365 PMC5944293

[39] Csizek Z Mikó-Baráth E Budai A Frigyik AB Pusztai Á Nemes VA Artificial intelligence-based screening for amblyopia and its risk factors: comparison with four classic stereovision tests. *Front Med*. (2023) 10:1294559. 10.3389/fmed.2023.1294559 38196833 PMC10775855

[40] Rauf N Gilani SO Waris A. Automatic detection of pathological myopia using machine learning. *Sci Rep*. (2021) 11:16570. 10.1038/s41598-021-95205-1 34400662 PMC8367943

[41] Koo S Kim WK Park YK Jun K Kim D Ryu IH Development of a machine-learning-based tool for overnight orthokeratology lens fitting. *Transl Vis Sci Technol*. (2024) 13:17. 10.1167/tvst.13.2.17 38386347 PMC10896231

[42] Fan Y Yu Z Peng Z Xu Q Tang T Wang K Machine learning based strategy surpasses the traditional method for selecting the first trial Lens parameters for corneal refractive therapy in Chinese adolescents with myopia. *Cont Lens Anterior Eye*. (2021) 44:101330. 10.1016/j.clae.2020.05.001 32418872

[43] Fan Y Yu Z Tang T Liu X Xu Q Peng Z Machine learning algorithm improves accuracy of ortho-K lens fitting in vision shaping treatment. *Cont Lens Anterior Eye*. (2022) 45:101474. 10.1016/j.clae.2021.101474 34301476

[44] Zhang M Guo Y Zhou C Zhang J Zhang M Huang J Deep neural network with self-attention based automated determination system for treatment zone and peripheral steepened zone in Orthokeratology for adolescent myopia. *Cont Lens Anterior Eye*. (2024) 47:102081. 10.1016/j.clae.2023.102081 37957085

[45] Lin WP Wu LY Li WK Lin WR Wu R White L Can AI predict the magnitude and direction of ortho-K contact lens decentration to limit induced HOAs and astigmatism? *J Clin Med*. (2024) 13:5420. 10.3390/jcm13185420 39336906 PMC11432668

