# Supplementary material for: Assessing large language models as assistive tools in selecting first trial lens parameters for orthokeratology
Source: Front Med (Lausanne). 2026 Feb 2;13:1741987. doi: 10.3389/fmed.2026.1741987 (PMC12907544; doi:10.3389/fmed.2026.1741987)
Supplement: Supplementary file 1 [file Data_Sheet_1.pdf]

Patient information is as follows

Age: 8 years old Gender: Male

Right eye refraction: -1.50DS/-0.50DC*180
Corrected visual acuity: 1.0
Axis length: 25.40 mm
Anterior chamber depth: 3.11mm
Corneal diameter: 11.67 mm
Corneal thickness: 568 µm
Corneal flat K: 41.15D Corneal steep K: 42.16D
Corneal E6: 0.55 Corneal E8: 0.58
Sag differential at 8 mm: 21µm

Left eye refraction: -1.75DS/-1.00DC*180
Corrected visual acuity: 1.0
Eye Axis Length: 25.56 mm
Anterior chamber depth: 3.11mm
Corneal Diameter: 11.85 mm
Corneal Thickness: 556 µm
Corneal flat K: 40.62D Corneal steep K: 42.02D
Cornea E6: 0.52 Cornea E8: 0.57
Sag differential at 8 mm: 21µm

1. What is the most likely diagnosis？
2. Does this patient need myopia prevention and control? Are orthokeratology lenses recommended as a means of prevention and control?
3. I would like to prefer Paragon CRT, please evaluate the patient's fitness by giving a diagnosis from the following 5 points: Corneal eccentricity, Astigmatism, Corneal diameter, Corneal curvature, Lens customization.
4. Please give the parameters of the first trial lens of Paragon CRT.

Re-evaluation of Accuracy and Revised Responses
The second inquiry: Some parameters are incorrect, please modify.
The third inquiry: Please refer to the CRT trial lens selection for the correct parameter combination.
